# Harmonizing disease prevention and police practice in the implementation of HIV prevention programs: Up-stream strategies from Wilmington, Delaware

**DOI:** 10.1186/1477-7517-9-17

**Published:** 2012-05-16

**Authors:** Basha Silverman, Corey S Davis, Julia Graff, Umbreen Bhatti, Melissa Santos, Leo Beletsky

**Affiliations:** 1 Brandywine Counseling and Community Services Inc, 2713 Lancaster Avenue, Wilmington, DE, 19805, USA; 2 Bryn Mawr Graduate School of Social Work, Social Work Research, 100 Airdale Road, Bryn Mawr, PA, 19010, USA; 3 Network for Public Health Law, 101 East Weaver Street, Suite G-7, Carrboro, NC, 27510, USA; 4 American Civil Liberties Union Foundation of Delaware, 100 W. 10th Street, Suite 603, Wilmington, DE, 19801, USA; 5 Santos Architecture and Graphic Design, 452 S. Atlantic Avenue, Pittsburgh, PA, 15224, USA; 6 Northeastern University School of Law, Bouve College of Health Sciences, 400 Huntington Ave, Boston, MA, 02115, USA

## Abstract

**Introduction:**

Improving access to sterile injection equipment is a key component in community-based infectious disease prevention. Implementation of syringe access programs has sometimes been complicated by community opposition and police interference.

**Case description:**

In 2006, the Delaware legislature authorized a pilot syringe exchange program (SEP). A program designed to prevent, monitor, and respond to possible policing and community barriers before they had a chance to effect program implementation and operation. A program designed to prevent, monitor, and respond to these barriers was planned and implemented by a multidisciplinary team of legal practitioners and public health professionals.

**Discussion:**

We report on an integrated intervention to address structural barriers to syringe exchange program utilization. This intervention employs community, police and client education combined with systematic surveillance of and rapid response to police interference to preempt the kinds of structural barriers to implementation observed elsewhere. The intervention addresses community concerns and stresses the benefits of syringe exchange programs to officer occupational safety.

**Conclusions:**

A cohesive effort combining collaboration with and educational outreach to police and community members based on the needs and concerns of these groups as well as SEP clients and potential clients helped establish a supportive street environment for the SEP. Police-driven structural barriers to implementation of public health programs targeting populations engaged in drug use and other illicit behavior can be addressed by up-stream planning, prevention, monitoring and intervention strategies. More research is needed to inform the tailoring of interventions to address police-driven barriers to HIV prevention services, especially among marginalized populations.

## Background

The spread of blood-borne disease through injection drug use is a longstanding problem, with a substantial proportion of HIV, hepatitis B, and hepatitis C (HCV) cases in the United States attributable to injection-related behaviors [1]. Evidence-based prevention interventions such as syringe exchange programs (SEPs) have been demonstrated to reduce risky injection-related behavior and injection-related transmission of blood-borne pathogens[2-5]. These programs also provide essential wrap-around services to marginalized drug users [6,7].

Police activity is an important structural determinant of SEP impact. Aside from direct police interference with program operations, [8-13] experience and perceptions of police practices can increase injection drug user (IDU) risk behavior [14-16] and deter uptake of SEP services [17-24]. Legal reform may be necessary, but it is generally not sufficient to align police practices with HIV prevention efforts [8,25,26]. The gaps in the “policy transformation process”[8] leave room for police officers to continue to confiscate legal injection equipment and interfere with the functioning of SEPs even where law has been changed to authorize these programs [26-29]. This may be because officers remain uninformed about laws decriminalizing SEPs or because they disregard them as misguided[26]. In some contexts, legalization of SEPs may promote arrest of SEP clients, possibly because of increased program visibility [28,30].

Unjustified and illegal police practices such as physical abuse of SEP clients, confiscation of legally-sanctioned injection equipment, uninvited appearances on program premises, and use of SEP participation as a marker of illegal behavior have been shown to deter SEP participation and contribute to unsafe injection practices [15,19,29,31-40]. Such actions may also undermine IDU trust in the public health laws designed to reduce disease risk and erode the credibility of risk reduction programs that promote safer behavior by advertising the legal protections afforded by public health-minded laws. The differential way in which racial/ethnic groups experience and perceive the law and law enforcement [41] may influence uptake of HIV prevention services, possibly contributing to the observed racial disparities in HIV incidence [17,18,25,42-44]. Furthermore, misalignment between public health and law enforcement efforts targeting drug users may adversely impact the occupational health of police and other criminal justice professionals, contributing in particular to elevated risk of needle-stick injury, [26,45] with possible impact on job stress, personnel burn-out and turnover[26,45,46]. There is some evidence, however, that under some circumstances police activity may promote drug user health when it is aligned with public health activities serving drug users [47].

Efforts to align police behavior with public health goals have been implemented elsewhere with positive results[48-50]. However, literature describing and evaluating these efforts remains sparse. This case study represents a case study of a comprehensive, public health prevention-focused approach to aligning police and public health activities targeting drug users in one US city.

## Case description: Prevention, monitoring and response

The role of law enforcement in the implementation of HIV-prevention efforts is a key arena in the growing movement to align policing and public health. The project presented here was designed as an integrated intervention to accompany the launch of a pilot SEP in Wilmington, DE, which operated under the auspices of authorizing legislation passed in 2006. Delaware’s relatively-late authorization of SEPs provided a unique opportunity to integrate lessons from elsewhere into a program to prevent, monitor, and respond to policing-related and community-driven barriers to SEP implementation and operation [29,43,46,47]. Under the terms of the law, the pilot project was permitted to operate only in the city of Wilmington and only through early 2012. The authors believed that success of the pilot SEP would be vital in arguing that the pilot program should be made permanent and expanded.

A collaborative team of legal practitioners from a non-profit civil rights organization and public health professionals planned and implemented a prevention, monitoring, and response (PMR) approach for public health-law enforcement collaboration.

Our goal was to assist a smooth roll-out of the pilot SEP initiative by pre-emptively addressing misinformation, distrust, and conflict between SEP staff and clientele and the local police department and addressing general community concerns regarding the SEP. The resulting program offers a modular toolkit for harm reduction programs and law enforcement organizations across the U.S., with possible application to international settings. The following section describes the specific aspects of the program design, including cross-references to the actual tools utilized in these efforts. Additional information about the toolkit is available on the Web at http://papers.ssrn.com/sol3/papers.cfm?abstract_id=1339323.

## Methods and tools

This project's methodology is based on the Rapid Policy Assessment and Response framework, an integrative action program that systematically engages local stakeholders in policy problem-solving [51]. To maximize the replicability of the PMR toolkit in other settings, we used a set of information technology tools that are freely available on the internet, including Google® documents, spreadsheets, and PDF maker.

## Program design

### Prevention

Some local groups, including members of the Wilmington Police Department, initially opposed SEP authorization. We began our efforts immediately following that authorization to attempt to dissipate this opposition to ensure that it would not compromise successful rollout of the SEP initiative. Our efforts were directed at achieving buy-in from and addressing concerns of three main groups 1) Community 2) Law Enforcement 3) IDUs.

#### Community

We held discussions with over 40 community groups in Wilmington, including neighborhood civic associations, community centers, and local small business owners, to build buy-in. We believed it was vital to have permission and validation from community residents who live in areas where the SEP would operate in order to eliminate service delivery barriers. We also believed that community buy-in would increase leverage with law enforcement. Our prevention efforts included educating community members on local disease prevalence and evidence of the effectiveness of SEPs. These discussions also provided community members an opportunity to have a voice in the program design and implementation. Concerns related to locations and hours of operation were incorporated into the final SEP design and implementation.

#### Law enforcement

Preliminary discussions with law enforcement administrators included concerns about police-related barriers to SEP implementation and officer needle stick injury (NSI) risk. We met with the Chief of the police department to discuss amending police standard operation procedures (SOPs) regarding syringe handling and disposal. Additionally, we were able to secure agreement that officers would undergo training on the specifics of the new SEP legislation, basic infectious disease information and contacts for where to address questions about SEP operations. Additionally, it was agreed that the SEP operator would work with the department’s Officer in Charge to hash out a number of unresolved practical questions, including the handling of syringes confiscated in the process of an inventory search of a SEP client who was being taken into custody. This collaborative effort led to the establishment of a tracking system that allows the police to avoid potential NSI by safely disposing of syringes collected during custodial inventory using biohazard receptacles provided by the SEP operator. In the place of the discarded needles, the client receives an official receipt they can later present to the SEP to receive a supply of syringes equivalent to what had been disposed of by police personnel.

#### Police training

Past research suggests that police do not always know or understand the policy behind SEPs and other harm reduction programs, do not readily discern the ways in which SEPs and other harm-reduction policy shapes their enforcement practice, and face heightened risk of NSI, which causes anxiety, apprehension and mistrust of IDUs[26,50]. Before the SEP was launched, the SEP operator approached the Wilmington police department with an offer to train street-level personnel on occupational safety issues including safe handling of syringes, the policy rationale behind the establishment of the program, and changes in the department’s standard operating procedures, amended to comply with the SEP legislation. The training was provided free of charge. Based on an existing level of good will that characterized its relationship with the SEP operator, departmental leadership acquiesced to this offer, recognizing that the training would fill an important gap in occupational safety and infectious disease education of department personnel.

SEP staff delivered a round of roll-call trainings to the entire police department. The training included information on the basic design of the SEP, its geographic scope, the authorizing legislation, and the legal immunities afforded to SEP clients. The curriculum stresses the public health goals of the program and the specific ways they shape the standard operating procedures of the department, including changes in search, arrest, and referral activities. It also describes the occupational safety procedures for handling syringes, communication techniques for prevention of NSI, and the appropriate actions to be taken in the case of an NSI. These trainings included a pre- and post-test evaluation to assess their effectiveness, based on previous models[49,50]. Participating officers received needle stick-resistant gloves as incentives for participation. Immediately after the completion of the course, the local SEP director worked with the police chief to secure permission from top-level management to train departmental staff to administer the training and agreement to add this training module to the standard coursework in the police academy. This permission was granted, and these trainings are ongoing. All incoming cadets in the department now receive the SEP training. In addition, police trainings have been provided to over 300 officers within the Wilmington, New Castle County, and Delaware State Police departments.

#### Police information card

**Figure 1:**
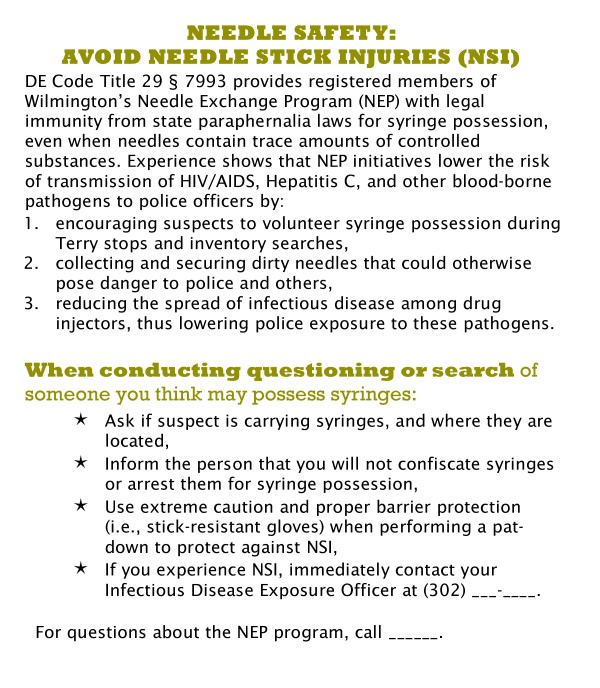
Police Wallet Information Card.

Training evaluations showed that officers may not retain all the communicated information, including occupational safety guidelines, standard operating procedures pertaining to SEP legislation, or logistics of SEP operations. It is important that street-level personnel have ready access to this information because it may impact their enforcement practices as well as their ability to properly direct questions and IDU referrals. In order to provide officers with this information in a user-friendly format, we created a wallet-sized information card (see Figure 1) which includes guidelines on avoiding NSI, information about the SEP authorization law, and information number officers can call with questions or referrals to the SEP. These cards are provided to each officer during the training.

#### Injection drug users

In order to inform the design of client-side informational and training resources, we conducted a focus group discussion with a group of current and former IDUs. Respondents were recruited from among drug treatment and HIV prevention program clients who were over 18 years of age and were not yet SEP clients. The script for the focus group discussion included the following topical areas: general and specific experiences with police and criminal justice system, perceptions of how police will respond to the operation of SEP, awareness of the changes in the law relating to the SEP, awareness of specific instances of arrest or harassment of SEP clients, critical analysis of existing components and materials of the PMR program, and ideas for additional resources the SEP can provide to increase their impact.

#### Know your rights (KYR) cards

**Figure 2:**
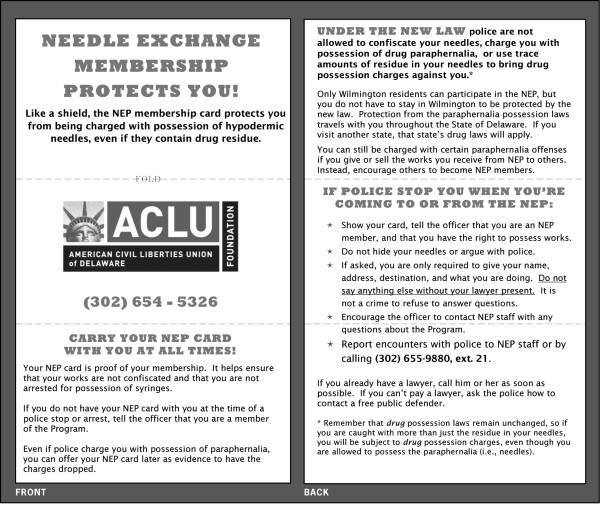
Client Know Your Rights Card.

Ensuring maximum SEP impact requires that clients fully understand and can avail themselves of the legal benefits of participation. Clients should have ready access to this information during an incident involving police contact. Knowing what to say, how to act, and where to direct questions they cannot answer can mitigate the effects of negative police interactions. In order to provide clients with this information in a user-friendly format, we created a wallet-sized information card (See Figure 2) for SEP clients, which includes information about the legal waiver the SEP law provides, general street law information, and a phone number that can be used to report an unfavorable incident with police or by police officers can call to confirm a client's membership or with questions about the operation of SEP. These cards were distributed to SEP staff, SEP clients, and to the attorneys of the Public Defender office.

#### IDU KYR training

Findings from a focus group demonstrated that IDUs-potential SEP clients--do not know or understand the legal protections extended to those participating in the SEP and fear that police will use the program to track their activities. Maximum SEP impact requires that potential clients understand that participation brings substantial legal protections. It is also key that members of the IDU community are educated about how the SEP legislation fits into the framework of their existing civil rights. To this end, we created and implemented Know-Your-Rights workshops for IDUs. Workshop participants were recruited from those enrolled in various programs at the SEP umbrella agency, which administers health and counseling services to IDUs. The 1 hour-long curriculum (developed in both English and Spanish) contained basic street law information, training on interacting with police officers, and specific information on the legal benefits flowing from SEP membership. The workshop also provided information on how to file grievances and to report incidents where the individual believes his or her rights were violated.

### Monitoring

#### Intake/exchange survey

Incidents of wrongful arrest, unlawful confiscation of injection equipment, and other negative interactions between potential and current SEP clients can discourage not only the affected individual from program participation but can quickly spread through informal networks, substantially endangering public health on the community level. However, unless they directly witness an incident or a client volunteers the information, program staff may not learn about client's negative experience with police. Robust surveillance mechanisms can help create a systematic way of collecting data about clients' experience with police, establishing an early-warning system and creating a source of data that can help inform police management decisions. We drafted 3 questions regarding arrest, confiscation of injection equipment, and interactions with police when en route to or from the SEP to capture a range of problematic experiences that may discourage SEP participation. These questions were coded into the hand-held computers the SEP staff uses to track client data. These questions about the client's experiences with police precede each intake and exchange transition. An indication that an incident of note has occurred will trigger the administration of an Incident Report (see below).

This early warning system helps alert SEP staff about adverse events and informs police management decisions. The following questions were asked at every instance of exchange, including time of enrollment, using an electronic tracking system.

1. In the last six months, how many times have you been arrested or cited for possession of syringes?

2. In the last six months, how many times have your works been confiscated without an arrest or citation?

3. In the last six months, how many times have you been stopped by police en route to or from the site?

#### Incident report

When SEP staff learn of an incident involving possibly inappropriate police conduct directed at a client or potential client, they need a systematic way of documenting the events. Surveillance tools such as the intake/exchange survey and outreach survey can capture the incidence of problems with law enforcement, but they do not provide sufficient detail to describe the nature of the individual incident. In-depth documentation is vital to discerning patterns of police activity, facilitating communication with police managers, and informing police management decisions. We created a 24-question form that prompts the respondent for details about the interaction with police, including logistical details, officer information, and circumstances surrounding the incident. SEP staff enter the information from these forms into an on-line database accessible to the SEPs legal partner: the Delaware chapter of the ACLU. Depending on the severity of the incident, this information may lead to immediate consultation with the legal partner or contact with the police department.

#### Public defender intake

Intake/exchange and outreach surveys are able to capture reports of IDU perceptions and experiences with police from only a limited contingent of IDUs. These surveillance mechanisms may not reach IDUs who are geographically removed from areas canvassed by exchange and outreach teams. To systematically capture law enforcement-related experiences within the wider IDU community, we advocated for the inclusion of SEP-specific questions into the standard intake process of the Public Defender's office. The questions prompt public defender IDU clients office for information about their arrest, police compliance with SEP legislation, and other key circumstances surrounding the incident.

#### Training

When IDUs are wrongfully arrested for possession of injection equipment or when they otherwise enter the criminal justice system, public defenders s and other criminal defense attorneys are often their first point of contact. These legal professionals have the duty to provide the most effective representation possible to their clients. Our preliminary research demonstrated that some public defenderss either did not know or did not understand the new state policy as it pertains to syringes. It is important to educate them about this new law because it may help them defend wrongfully-arrested individuals. These providers can also help discern patterns in police enforcement and help sound a bell of alarm when certain repeating fact patterns begin appearing before them in court. To this end, we designed a 1/2 hour continuing legal education workshop, focusing on policy and research evidence supporting SEPs, operational details of the SEP program, legal implications of the new law, and documentation and referral information as it pertains to cases of police misconduct relating to IDUs. The workshop was attended by 20 public defender attorneys and may be repeated in the future.

#### Phone hotline

A local telephone line at the SEP was designated for fielding and collecting client reports of incidents with police. The line includes 24-hour voice messaging functionality. The SEP program director fields the calls and documents any incidents using the Incident Report Form. The hotline was publicized through KYR cards, police cards, SEP fliers, and other materials.

### Response

#### Police liaison

Open lines of communication help identify problems early and address them by using management and training instead of litigation. However, before the pilot program was authorized, the Wilmington Police Chief spoke out adamantly against the initiative: “No matter how you look at this issue, both sides would have to agree that it boils down to putting clean needles in the hands of the addicted so they can continue their illegal and dangerous activity”[52]. Because the legislation authorizing the SEP initiative required the new program to coordinate its efforts with police, the Chief appointed a liaison officer whom SEP program staff can contact with information about police failing to follow the SEP authorization law. In order to ensure early detection and response, regular meetings are held between the SEP and its legal partner organization. When adverse events do occur, SEP and the police liaison can rely on surveillance and incident report data in deciding what action is needed. Based on surveillance and incident report data, the SEP operator or police department can decide whether and how to address the problem. The range of options at the police department level includes additional training, change of assignment, or disciplinary action against the officer.

#### Legal action

As a last resort, the SEP and its legal partner can use collected data to pursue administrative or legal action against the department and/or the officer involved. However, experience elsewhere suggests that prevention tends to be much more effective and efficient than adversarial action after-the-fact [6].

## Discussion

Preliminary data suggests that the project had a positive impact. Early investment in engaging community and police in program planning created a positive working environment as the program has expanded. More than five years after the program’s launch, SEP staff has reported only 12 incidents of police harassment or interference with their activities. Compared with the experience nationally, this is an encouraging outcome [25].

Each of the 12 incident reports have been followed-up by contacting our police liaison. Each incident of client arrest for syringe possession was discussed with the police liaison. Follow-up discussion offered SEP staff an opportunity for education and reinforcement of new policy. Each incident report resulted in the SEP staff contacting the public defender office to ensure the charges were dropped. SEP staff were able to verify the date of the participant’s enrollment and participation in the syringe exchange program.

One of the most telling facets of the Wilmington story was that, two years after the police Chief’s original statement on the SEP initiative, Chief Szczerba announced “My opinion of the program is no longer relevant, but the success of this program is. […] I'm committed to providing leadership and cooperation from the law-enforcement end”[53]. This was done following the concerted SEP relation-building efforts with police and members of the community. In addition, the relation-building efforts have paved the way for forging ties with other law enforcement agencies including the Department of the State Attorney General, who has contacted the program on several occasions to inquire about the implementation of the syringe legislation.

In July 2011 a new law was passed moving the SEP from pilot to permanent status. This achievement would not have been possible without evidence of program effectiveness as a reliable and cost-effective public health intervention as well as increased support from local law enforcement.

### Strengths and limitations

There have been additional benefits to the SEP. Police trainings gave SEP staff opportunity to build better relationships with the department, putting a face to SEP practitioners. For example, Delaware’s legislation includes a clause that requires an Oversight Committee be formed and meet quarterly to monitor implementation and make minor changes to SOPs to improve programming within the limits of the law. The City of Wilmington’s Chief of Police is a member of that Oversight Committee. As a result of this project, the Chief of Police was impressed with the SEP staff commitment to officer safety. The Chief created a Law Enforcement Oversight subcommittee whose membership was made up of the Director of the SEP, the designated police liaison, community police officers, and representatives from the Delaware Division of Public Health. These meetings created an opportunity for public health practitioners and law enforcement to work together to discuss obstacles, best practices, and concerns about each agency meeting their respective needs. These opportunities for relationship building, which included these regular meetings as well as routine training opportunities, strengthened the relationship between social service providers and the criminal justice system.

In addition to SEP staff participation in the Law Enforcement Subcommittee meetings and SEP having a single point of contact within the police department, SEP staff have established working relationships with community police officers who are assigned to much more limited areas within the city. These community police officers know the SEP staff most intimately, offering protection to SEP staff and referring potential SEP participants to the SEP services. Police have referred at least 10 participants to the program to date. SEP staff have received positive feedback about the KYR cards both from the clients and the police.

The public defender training provided a relationship-building opportunity between SEP staff and the public defender office. The SEP staff receive phone calls from the public defender office to verify client enrollment whenever necessary. The participants of the exchange recognize that SEP staff monitor their police involvement and respond to incident reports. These SEP practices have likely contributed to trust in the program among participants and facilitated utilization of SEP services.

Community civic associations are vital partners in the effort to prevent bloodborne disease infection. The SEP authorization law includes a requirement that the SEP be mobile, operating within Wilmington in high-risk neighborhoods. SEP staff recognized the importance of community buy-in before start up. SEP staff identified a list of existing civic associations in the proposed SEP service area, contacted the president of each organization, and asked for an invitation to attend the next organizational meeting. SEP staff attended a total of 16 civic association meetings, presenting information on disease transmission, local disease prevalence and the effectiveness of SEP. In addition, SEP staff asked the civic association members for their permission to park the mobile program unit in their neighborhood. Members expressed concerns related to specific locations and hours of operation, citing daycare, traffic, and youth walking home from school. SEP staff honored these requests and designed a schedule that met their recommendations but was still able to effectively meet the needs of SEP clients. Since then, SEP staff return to these regular meetings every 4–6 months to give the members an updated report on the program results. Community civic association support has been vital to the continuation of this program and was integral in a recent legislative change making the pilot project permanent.

Constant and consistent communication between police and SEP staff have contributed to the sense of safety and protection for SEP staff. Since the program was launched, there have been no incidents involving police harrassment of SEP staff; instead, police have contacted the staff on several occasions to alert them about possible dangerous activity unfolding in the areas of the mobile exchange. Although no precise cost analysis is feasible, the reduction in arrest and syringe confiscation reported by clients suggests significant cost savings to the criminal justice system.

SEP program staff were forced to make modifications to our original design, as well as utilize resources which were unanticipated but proved to be necessary. We purchased needle resistant gloves for every patrolling officer in the department. We also provided sharps containers to each officer and installed a large disposal receptacle in each station. We make routine visits to the stations to remove filled containers and install replacements. We believe these investments are more than justified given their importance in relationship building between the SEP provider and police department.

Despite the successes, a number of challenges remain: Not all program components could be implemented as planned. The outreach surveys designed to be distributed by SEP outreach workers to non-SEP participants were difficult to complete in the street setting. Because of lack of resources, outreach workers were unable to dedicate time and resources necessary to conduct these surveys during the hours they were dedicated to providing services on the SEP mobile van. Additional human and financial resources and recruitment incentives would help to implement this monitoring scheme to track experiences and attitudes of the non-client IDU community.

The intake questionnaire designed for the Public Defender office was not adopted and we had no authority to enforce its utilization.

Incident reports provide both benefits and drawbacks. They add to the client perception that SEP cares enough to document police encounters in detail. They also provide a tool for SEP to follow up with police. However, clients rarely possess or are willing to provide the full array of information requested.

While police training opportunities exist, time for these workshops is limited. Police work is typically based on shifts and training opportunities for the entire department are rare and happen only one to two times per year. We have been forced to modify our training and to create a shorter version to be used during roll call opportunities. These 15–25 minute brief trainings were offered to police during their “roll calls,” short meetings convened by police administration to brief officers before their shifts begin. The SEP Program Director has completed 55 roll call trainings. These roll call trainings are completed multiple times per day on a routine basis, every 3 months. They take time and energy from already stretched SEP resources. We have also conducted 7 1.5 hour trainings with department staff during organized in-service trainings, and our training has also been added to the Police Academy curriculum.

## Conclusion

Cross-sectoral collaboration has proven valuable. We believe a good working relationship with local law enforcement was key to the success of the pilot SEP and was an important factor of the recent decision by the legislature to make the pilot program permanent. SEP staff hold regular meetings and cellular phone contact with police leadership, which is now defending the program against NIMBY efforts [11]. Patrolling officers join SEP staff at community meetings. Police make referrals to the SEP, although perhaps not as often as we would like.

Among implementation challenges, most significant was the curtailing of the outreach survey. Based on Brandywine Counseling annual client census data, utilization of methadone programs by Black IDUs in Wilmington is more than twice than the utilization of the SEP, suggesting that barriers specific to this group discourage SEP participation. More research is needed to assess the accuracy of anecdotal reports, supported by findings from our discussions with IDUs, that this disparity is driven by differential perceptions and experiences of the criminal justice system. The reluctance to access evidence-based HIV prevention services may partially explain stark disparities in HIV incidence among African-Americans. More research and resources are needed to accurately assess the existence and etiology of this racially-disparate risk environment in order to improve the design and dosage of structural interventions that improve program access.

This investment in time and financial resources has positive effects that can be seen beyond uptake of SEP services. The fact that the SEP provider has such a positive and consistent relationship with law enforcement has helped to legitimize the SEP. The local health department boasts about this to other agencies. Both SEP staff and the police have come to recognize that our mission is the same - to help keep our community members healthy and safe - we just have different way of approaching it.

## Competing interests

No author received direct financial benefit from any product or service described.

## Authors’ contributions

LB, JG, and BS conceptualized the project and secured the funding. LB and JG designed the operational strategy. BS conducted community meetings. CD and MS helped design program materials. LB prepared the first draft of the manuscript, which was completed by BS and edited by CD with input from all authors. BS served as the SEP operator. JG, UB and BS completed the CLE trainings. BS completed all police trainings. All authors contributed to and reviewed the manuscript.
